# Acute macular neuroretinopathy with coexistent central retinal vein occlusion as the presenting feature in intraocular tuberculosis

**DOI:** 10.1186/s12348-020-00201-7

**Published:** 2020-02-27

**Authors:** Ramesh Venkatesh, Sajjan Sangai, Arpitha Pereira, Padmamalini Mahendradas, Naresh Kumar Yadav

**Affiliations:** Department of Retina, Vitreous and Ocular Inflammation, Narayana Nethralaya, #121/C, 1st R block, Chord Road, Rajaji Nagar, Bengaluru, 560010 India

**Keywords:** Intraocular tuberculosis, Acute macular neuroretinopathy, Central retinal vein occlusion

## Abstract

**Aim:**

To report a case of intraocular tuberculosis presenting as acute macular neuroretinopathy and central retinal vein occlusion.

**Case description:**

A 29-year-old man presented to the retina clinic with complaints of sudden blurring of vision in the left eye of 3 days duration. His visual acuity was 6/6 and 6/18 in the right and left eye, respectively. Fundus examination of the left eye showed features of central retinal vein occlusion. OCT showed features of type 2 acute macular neuroretinopathy (AMN) as well. Over a period of 2 weeks, the patient developed choroidal granulomas with overlying retinal elevation and peripapillary choroidal neovascular membrane and retinal granuloma. Mantoux test and HRCT chest confirmed the diagnosis of pulmonary tuberculosis.

**Results:**

The patient was treated with a course of antitubercular therapy, oral corticosteroids and a single dose of intravitreal anti-vascular endothelial growth factor (1.25 mg/0.05 ml Bevacizumab, Roche Pharma) injection. After 6 months of therapy with ATT and tapering course of oral steroids, there was a complete resolution of all clinical signs including the choroidal granuloma with an improvement in visual acuity to 6/6.

**Conclusion:**

Acute macular neuroretinopathy can complicate intraocular TB. Tuberculosis should be kept as one of the differential diagnosis in patients with AMN. Prognosis is generally good in patients of ocular TB presenting with retinal vascular occlusions.

## Background

Ocular tuberculosis (TB) presents with a variety of intraocular clinical features like conjunctival granuloma, granulomatous anterior uveitis, multifocal choroiditis, chorioretinitis, choroidal granulomas and retinal vasculitis [1, 2]. Periphlebitis is a common manifestation of TB uveitis and was known earlier as Eales’ disease. Tubercular retinal vasculitis initially presenting as the central or branch retinal vein or artery occlusions with or without other ocular signs of TB has been rarely described previously [3–7]. Acute macular neuroretinopathy (AMN) was described by Bos and Deutman in 1975 as a characteristic red, wedge-shaped defect affecting the macular inner retinal layers [8]. With the more sophisticated imaging systems, such as spectral-domain optical coherence tomography (SD-OCT), Sarraf et al. classified AMN into 2 types: (a) type 1 AMN, where hyperreflective band is noted in the outer plexiform layer/inner nuclear layer region with subsequent inner nuclear layer thinning and (b) type 2 AMN, where hyperreflective band is seen in the outer plexiform layer/outer nuclear layer region with subsequent outer nuclear layer thinning and concomitant defects of the inner segment/outer segment layer [9]. Later, type 1 AMN came to be known as paracentral acute middle maculopathy and type 2 AMN as acute macular outer retinopathy. Patients with AMN complain of sudden blurring of vision with paracentral scotoma usually in the one eye. The diagnosis of AMN is confirmed by characteristic imaging features on SD-OCT. Analysis of risk factors in AMN seems to suggest a retinal microvascular aetiology [10]. Ocular TB as a cause of AMN has not been described in the literature to the best of our knowledge. In this report, we describe a case of ocular TB presenting with combined central retinal vein occlusion and type 2 AMN and treated successfully with antitubercular therapy (ATT) and oral corticosteroids.

## Case report

**Fig. 1:**
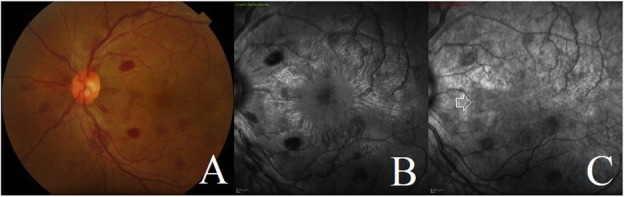
Conventional colour fundus photograph and multicolour imaging of the left eye at presentation. **a** Conventional colour fundus photograph of the left eye showing the multiple flame-shaped and deep retinal haemorrhages spread across the retina along with dilated and tortuous retinal veins suggestive of central retinal vein occlusion. **b** Thirty degrees of green reflectance image showing the dark hyporeflectance patches corresponding to the retinal haemorrhages. **c** Thirty degrees of infrared reflectance image showing a diffuse patch of hyporeflectance involving the nasal, superior and inferior macular quadrants corresponding to the AMN lesion (white arrow). The retinal haemorrhages appear less dark on infrared reflectance compared to the green reflectance image

**Fig. 2:**
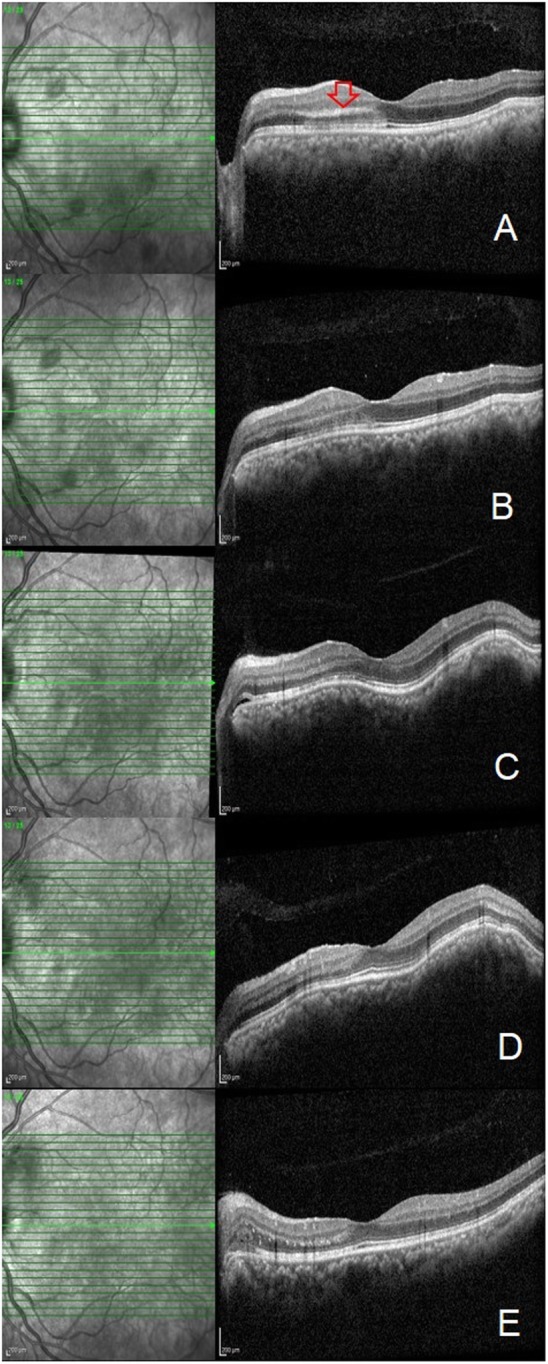
Sequential OCT scans of the left eye at presentation, 4-day, 7-day, 14-day, and 6-month visits. **a** Horizontal raster OCT scan at presentation showing a hyperreflective plaque at the outer side of the outer plexiform layer with hyperreflectivity of the outer nuclear layer and disruption of ellipsoid zone involving the nasal macular quadrant suggestive of type 2 AMN (red arrow). **b** Horizontal raster OCT scan at 4 days showing reduced hyperreflectivity and resolution of the AMN lesion. **c** OCT scan at 7 days showing RPE elevation by the underlying choroidal lesion (white arrow). **d** OCT scan at 14 days post presentation showing progressive increasing RPE elevation due to the choroidal granuloma (white arrow). **e** OCT scan at 6-month post ATT showing resolution of the choroidal granuloma (white arrow)

**Fig. 3:**
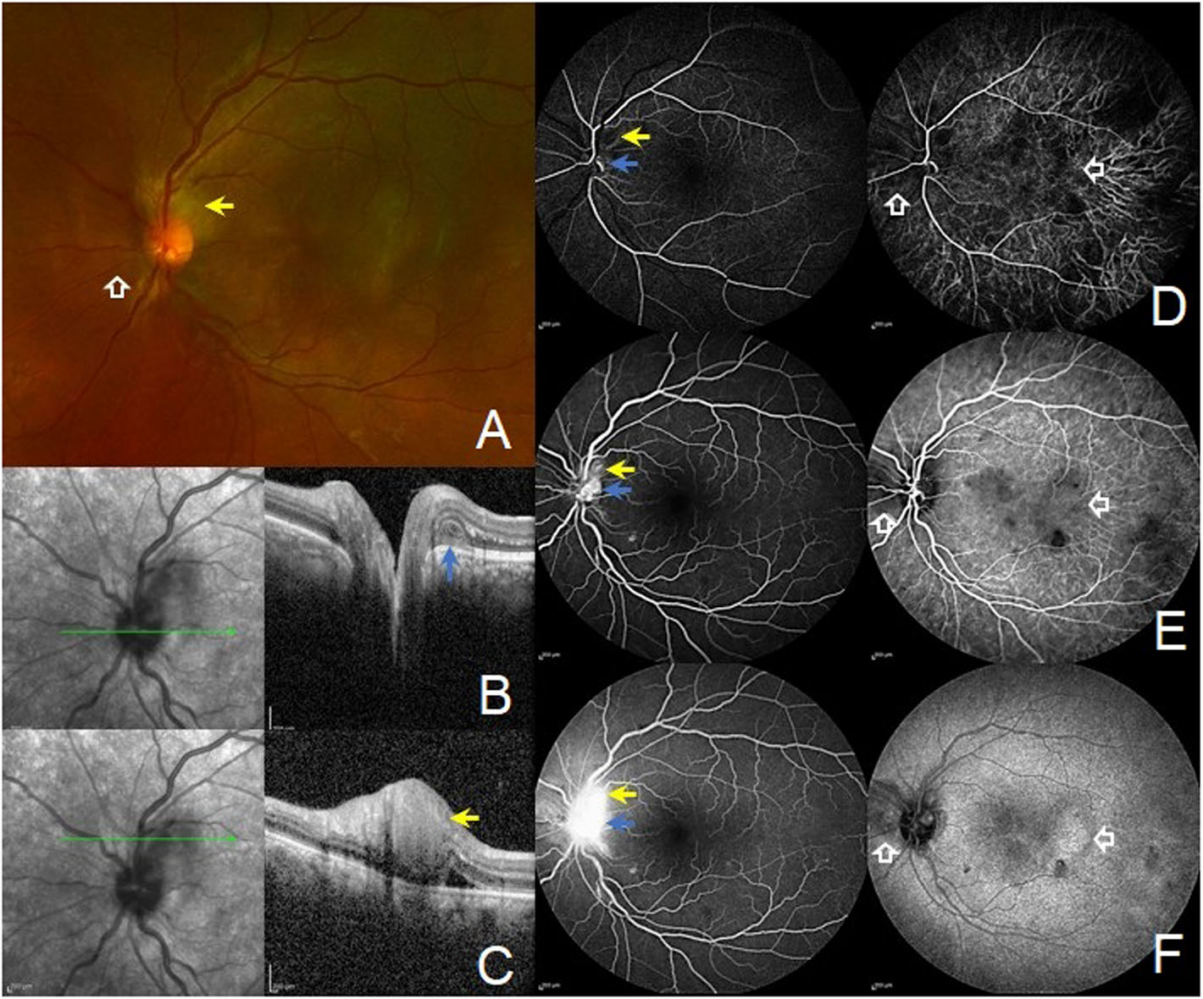
Multimodal imaging findings revealing the presence of retinal and choroidal granulomas and peripapillary choroidal neovascular membrane (CNV). **a** The pseudocolour image with Optos, Daytona shows a grey-white lesion superotemporal to the optic nerve head (yellow solid arrow) and subtle elevated lesion nasal to the optic disc and temporal to the macula (white hollow arrows) suggestive of choroidal granulomas. **b** Horizontal line scan through the middle portion of the optic disc reveals the presence of peripapillary, subretinal choroidal neovascular membrane (blue solid arrow). **c** Horizontal line scan through the retina superior to the optic disc shows the presence of granuloma within the retinal layers (yellow solid arrow) and associated subretinal fluid. **d**–**f** Progressive phases of combined fluorescein (FA) and indocyanine green angiography (ICGA) of the left eye. The retinal granuloma, seen superotemporal to the optic nerve head, shows hypofluorescence in early phase with increasing hyperfluoroscence in the late phases of the FA (yellow solid arrow). On the ICGA, the lesion shows early hypofluorescence with mild staining of the lesion in the late phases (yellow solid arrow). The choroidal granulomas are not identified on the FA. The choroidal granulomas are seen on the ICGA as early hypofluorescent lesions with staining in the late phases (white arrow). The middle portion of the optic nerve head identifies a mild hyper fluorescent lesion in the early phase of the FA which increases in intensity and size in the late phases suggestive of peripapillary CNV (blue solid arrow). Capillary non-perfusion areas are not visualised on FA. On the ICGA, the neovascular complex involving the optic nerve head is well delineated (blue solid arrow)

A 29-year-old man presented to the retina clinic with complaints of sudden blurring of vision in the left eye of 3 days duration. He was not a known diabetic or hypertensive and had no significant treatment history. His presenting visual acuity in the right eye (RE) and left eye (LE) was 6/6 and 6/18, respectively. Anterior segment examination and intraocular pressure in both eyes were normal. Fundus examination of the RE was normal. Fundus examination of the LE showed multiple blot- and flame-shaped haemorrhages with markedly dilated retinal veins in all quadrants. Macula showed no visible changes (Fig. 1). A clinical diagnosis of central retinal vein occlusion (CRVO) in LE was made. His blood pressure on examination was 140/80 mm Hg. Multimodal imaging was done which included conventional and pseudo-colour fundus photography (CFP), multicolour imaging and OCT (Spectralis, Heidelberg Engineering, Heidelberg, Germany). Horizontal line OCT scan passing through the macula showed a hyperreflective plaque at the outer side of the outer plexiform layer with hyperreflectivity of the outer nuclear layer and disruption of the ellipsoid zone involving the nasal, superior and inferior macular quadrants. No intraretinal fluid was seen (Fig. 2). These features on OCT suggested an associated type 2 AMN as well. He was advised for haematological investigations including serum homocysteine levels, Mantoux test and chest high-resolution computed tomography (HRCT) scans. Patient followed-up after 1 week with reports. Routine haematological investigations were normal including the serum homocysteine levels (15.6 μmol/L; range 5–16 μmol/L). Positive Mantoux test was noted with induration size measuring 15 × 15 mm with 5 TU PPD. HRCT chest showed an area of interlobular septal thickening involving the right middle lobe lateral segment and right and left basal segments. Few tiny nodules involving the right posterior basal segment were identified as well. These findings were suggestive of pulmonary TB. One week after presentation, the left eye fundus showed a subtle choroidal elevation temporal to the macula suggestive of choroidal granuloma. Based on the ocular findings, radiological and immunological evidences, a diagnosis of probable intraocular TB presenting with combined CRVO and type 2 AMN was made. The patient was referred to the pulmonologist to start treatment with ATT and obtain clearance for oral corticosteroids. He followed-up 2 weeks later with the left eye fundus showing multiple increasing choroidal granulomas. Fluorescein and indocyanine green angiography were done which delineated the location and extent of these tubercular choroidal granulomas. Also, extensive leakage at the disc and peripapillary region was noted due to inflammation from active peripapillary retinal and choroidal granuloma and presence of peripapillary choroidal neovascular membrane (CNV) (Fig. 3). The patient was started on treatment with oral ATT and corticosteroid therapy. A single dose of intravitreal anti-vascular endothelial growth factor (1.25 mg/0.05 ml Bevacizumab, Roche Pharma) injection was given for the peripapillary CNV. After 6 months of therapy with ATT and tapering course of oral steroids, there was a complete resolution of all clinical signs including the choroidal granuloma with improvement in visual acuity to 6/6.

## Discussion

A variety of clinical signs are seen in patients with ocular TB; however, retinal vascular occlusion alone is rare and only a few cases have been reported in the literature [3, 5–7, 11]. To the best of our knowledge, AMN along with CRVO as a presenting feature in ocular TB has not been reported. Although the pathogenesis of AMN is complex, recent research suggests ischemia to the retinal deep capillary plexus. There are various theories for retinal vascular occlusion in ocular TB: [1] direct vessel blockage by M. tuberculosis, [2] compression from a retinal or choroidal tubercle and [3] disc swelling associated with inflammation caused by tuberculosis infection and hypersensitivity to M. tuberculosis.

In our case, the features of retinal vascular occlusion resolved completely after starting the patient on a course of ATT and oral systemic steroids. After starting treatment, there was resolution of retinal haemorrhages and choroidal elevation on clinical examination and OCT scans showed resolution of choroidal granuloma. Several similar reports have demonstrated excellent results in the eyes with retinal vascular occlusion secondary to TB with treatment with ATT alone in the absence of other clinical features of ocular TB [3, 5–7]. Adjunctive tapering dose corticosteroid therapy was given in our patient to prevent any ocular tissue damage due to delayed hypersensitivity to tubercular antigen. In our case, we performed the treatment with one injection of intravitreal Bevacizumab for the treatment of inflammatory peripapillary CNV. The CNV had regressed at the follow-up visit.

## Conclusion

To conclude, AMN can complicate intraocular TB. Prognosis is generally good in patients of ocular TB presenting with retinal vascular occlusions.

## Data Availability

The datasets used and/or analysed during the current study are available with the corresponding author on reasonable request.

## Abbreviations

AMN: Acute macular neuroretinopathy
ATT: Antitubercular therapy
CFP: Colour fundus photography
CNV: Choroidal neovascular membrane
CRVO: Central retinal vein occlusion
HRCT: High-resolution computed tomography
OCT: Optical coherence tomography
TB: Tuberculosis

## References

[1] Cutrufello NJ, Karakousis PC, Fishler J, Albini TA (2010). Intraocular tuberculosis. Ocul Immunol Inflamm..

[2] Teixeira-Lopes F, Alfarroba S, Dinis A, Gomes MC, Tavares A (2018). Ocular tuberculosis - a closer look to an increasing reality. Pulmonology..

[3] Fullerton DG, Shrivastava A, Munavvar M, Jain S, Howells J, Macdowall P (2007). Pulmonary tuberculosis presenting with central retinal vein occlusion. Br J Ophthalmol..

[4] Gupta A, Gupta V, Arora S, Dogra MR, Bambery P (2001). PCR-positive tubercular retinal vasculitis: clinical characteristics and management. Retina Phila Pa..

[5] Mahyudin M, Choo MM, Ramli NM, Omar SS (2010). Ocular tuberculosis initially presenting as central retinal vein occlusion. Case Rep Ophthalmol..

[6] O’Hearn TM, Fawzi A, Esmaili D, Javaheri M, Rao NA, Lim JI (2007). Presumed ocular tuberculosis presenting as a branch retinal vein occlusion in the absence of retinal vasculitis or uveitis. Br J Ophthalmol..

[7] Ooi YL, Tai LY, Subrayan V, Tajunisah I (2011). Combined optic neuropathy and central retinal artery occlusion in presumed ocular tuberculosis without detectable systemic infection. Ocul Immunol Inflamm..

[8] Bos PJ, Deutman AF (1975). Acute macular neuroretinopathy. Am J Ophthalmol..

[9] Sarraf D, Rahimy E, Fawzi AA, Sohn E, Barbazetto I, Zacks DN (2013). Paracentral acute middle maculopathy: a new variant of acute macular neuroretinopathy associated with retinal capillary ischemia. JAMA Ophthalmol..

[10] Bhavsar KV, Lin S, Rahimy E, Joseph A, Freund KB, Sarraf D (2016). Acute macular neuroretinopathy: a comprehensive review of the literature. Surv Ophthalmol..

[11] Yuksel E, Ozdek S (2013). Unusual presentation of ocular tuberculosis: multiple chorioretinitis, retinal vasculitis and ischaemic central retinal vein occlusion. Clin Exp Optom..

